# A Metric of Influential Spreading during Contagion Dynamics through the Air Transportation Network

**DOI:** 10.1371/journal.pone.0040961

**Published:** 2012-07-19

**Authors:** Christos Nicolaides, Luis Cueto-Felgueroso, Marta C. González, Ruben Juanes

**Affiliations:** 1 Department of Civil and Environmental Engineering, Massachusetts Institute of Technology, Cambridge, Massachusetts, United States of America; 2 Engineering Sciences Division, Massachusetts Institute of Technology, Cambridge, Massachusetts, United States of America; 3 Center for Computational Engineering, Massachusetts Institute of Technology, Cambridge, Massachusetts, United States of America; Northeastern University, United States of America

## Abstract

The spread of infectious diseases at the global scale is mediated by long-range human travel. Our ability to predict the impact of an outbreak on human health requires understanding the spatiotemporal signature of early-time spreading from a specific location. Here, we show that network topology, geography, traffic structure and individual mobility patterns are all essential for accurate predictions of disease spreading. Specifically, we study contagion dynamics through the air transportation network by means of a stochastic agent-tracking model that accounts for the spatial distribution of airports, detailed air traffic and the correlated nature of mobility patterns and waiting-time distributions of individual agents. From the simulation results and the empirical air-travel data, we formulate a metric of influential spreading––the geographic spreading centrality––which accounts for spatial organization and the hierarchical structure of the network traffic, and provides an accurate measure of the early-time spreading power of individual nodes.

## Introduction

The study of complex systems as networks has revolutionized many disciplines in physics and the social and natural sciences [1]–[4]. The spreading of infectious diseases is an important example that illustrates the societal impact of global connectivity in man-made transportation systems [5], [6]. Outbreaks expose the vulnerability of current human mobility systems, and challenge our ability to predict the likelihood of a global pandemic, and to mitigate its consequences [7].

Network models of epidemic spreading have rationalized our understanding of how diseases propagate through a mobile interactome like the human population. “Fermionic” models regard each node as an individual, or a perfectly homogeneous community. In these models, the epidemic threshold for disease spreading vanishes in (infinite-size) scale-free networks, owing to the broad degree distribution [8], [9]. “Bosonic”, or metapopulation, models conceptualize nodes as subpopulations that can be occupied by a collection of individuals [10], [11]. Metapopulation network models thus recognize that spreading of a disease within a node is not instantaneous. Here we adopt a metapopulation-network approach, precisely because of the interacting timescales for traffic-driven transport between nodes and contagion kinetics within nodes.

It has been shown recently that advection-driven transport, or bias, in complex networks exerts a fundamental control on agent spreading [12], leading to anomalous growth of the mean square displacement, in contrast with purely diffusive processes. The crucial role of traffic-driven transport has also been pointed out in the context of epidemic spreading [13], where it has been shown to directly affect epidemic thresholds.

Given that epidemic spreading is mediated by human travel, and that individual human mobility is far from being random [14]–[16], it is natural to ask how the non-Markovian nature of individual mobility affects contagion dynamics. A model of recurrent mobility patterns characterized by a return rate to the individual’s origin has recently been incorporated into an otherwise diffusive random-walk metapopulation network model [17], [18]. A mean-field approximation, as well as Monte Carlo agent-based simulations of the process, reveal a transition separating global invasion from extinction, and show that this transition is heavily influenced by the exponent of the network’s degree distribution [17].

The impact of behavioral changes on the invasion threshold and global attack have recently been analyzed in the context of an SIR infection model [19]. In that study it is shown how individual re-routing strategies, where individuals modify their travel paths to avoid infected nodes, influence the invasion threshold and global levels of infection. It is found that selfish individual behavior can have a detrimental effect on society as a whole by inducing a larger fraction of infected nodes, suggesting that the concept of *price of anarchy* in transportation networks [20] operates also during disease spreading at the system level.

Taken together, these previous results reflect an emphasis on the asymptotic late-time behavior of contagion processes, typically characterized by infection thresholds and the fraction of infected nodes for both “fermionic” [13], [21] and “bosonic” networks [10], [11], [17], [19], but leave open the question of what the early-time behavior is [22]. Here, we address this question by developing a framework for contagion dynamics on a metapopulation network that incorporates geographic and traffic information, as well as the time-resolved collective transport behavior of individual stochastic agents that carry the disease. Resolving the temporal dynamics is critical to capture the nontrivial interplay between the transport and reaction timescales.

In this article, we present a new metric to identify and rank influential spreaders of infectious diseases in human transportation networks. Our metapopulation model of contagion dynamics is based on a time-resolved stochastic description of individual agent mobility through the air transportation system. The model is traffic-driven, and agents traverse the network following empirical stochastic rules that reflect the patterns of individual human mobility [15], [16]. These rules include exploration and preferential visit [16], and distributions of waiting times between successive flights that depend on demography. We show that the late-time spreading, as measured by the global attack, depends strongly on traffic and heterogeneity of transition times. We are interested in characterizing, *a priori*, the early-time spreading potential of individual nodes, as measured by the total square displacement of infected agents. We find that existing metrics of influential spreading––including connectivity [1], betweenness centrality [23] and 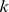-shell rank [24]––do not successfully capture the spreading ability of individual nodes, as revealed by Monte Carlo simulations. We show that the origin of this disparity lies on the role of geography and traffic on the network [25], and we propose a new metric––the geographic spreading centrality––tailored to early-time spreading in complex networks with spatial imbedding and heterogeneous traffic structure.

## Results

### Stochastic Model of Agent Mobility

#### Air transportation data

**Figure 1:**
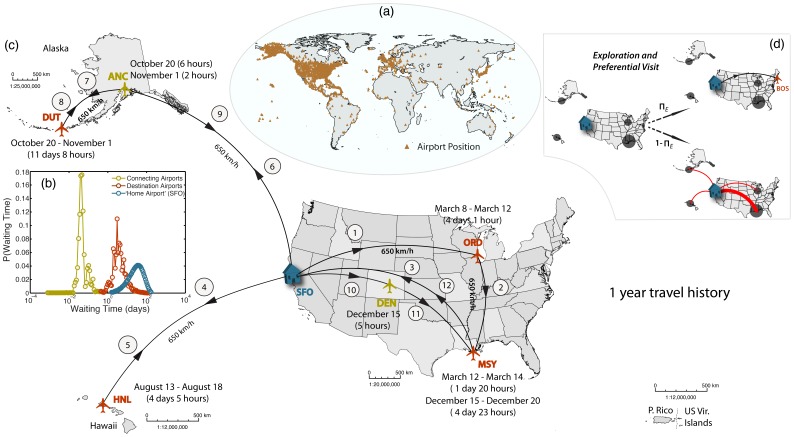
Pictorial view of the key elements of our empirical model of human mobility through the air transportation network. (*a*) World map with the location of the 1833 airports in the US database from the Federal Aviation Administration (www.faa.gov). (*b*) Waiting time distributions at connecting and destination airports (from [26]), and at the “home” airport. (*c*) Illustration of a 1-year travel history of an individual with “home” at San Francisco International Airport (SFO). (*d*) Graphical representation of the probabilities for exploration and preferential visit of the same individual, after the 1-year “training period.” During exploration the agent visits a new airport while during preferential visit the agent visits a previously-visited place with probability proportional to the frequency of previous visits to that location.

We develop a stochastic model of human mobility through a US-centric air transportation network. We use air-travel data provided by the Federal Aviation Administration (www.faa.gov) that includes all flights from all domestic and international airlines with at least one origin or destination inside the US (including Alaska and Hawaii), for the period between January 2007 and July 2010. Note that we do not have traffic information about flights whose origin and destination is outside the US. The air transportation network is a space-embedded network with 1833 airports, or nodes, and approximately 50,000 connections, or directed links (Fig. 1*a*). It is a highly heterogeneous network with respect to the degree 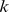 (or connectivity) of each node, the population associated with each node, as well as the traffic volume through the links of the network [13], [23]. The traffic data is organized in two datasets: “Market” and “Segment”. The Market dataset counts trips as origin-to-final-destination, independently of the number of intermediate connecting fights. The Segment dataset counts passengers between pairs of airports, without consideration of the origin and final destination of the whole trip. For example, a passenger that travels from Boston (BOS) to Anchorage (ANC), with connecting flight at Seattle (SEA), would be counted only once in the Market dataset as a passenger from BOS to ANC. In the Segment dataset, however, the passenger would be counted both in the segment BOS-SEA, and in the segment SEA-ANC. From these datasets we extract two weighted matrices that characterize the network traffic: a traffic flux matrix 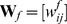 where 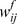 is the yearly passenger traffic from origin 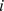 to destination 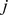; and a traffic transport matrix 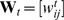 where 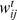 is the yearly passenger traffic in the segment from airport 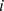 to airport 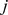.

In addition to the aggregate traffic data, we use information of individual itineraries, provided by a major US airline for domestic trips [26]. This dataset extends over a period of four months in 2004 and includes 3.2 million tickets. We use it to extract the waiting time distribution at final destinations and at connecting airports (Fig. 1*b*).

#### Empirical model

We use the data to build an empirical model of human mobility through the air transportation network. To each airport 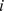, we assign a population 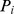 by an empirical relation [27], 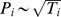, which reflects a correlation between population and yearly total outgoing traffic at that airport, 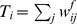. Therefore, each individual agent in the model has a “home airport” [17], [19].

Individual agents traverse the network following empirical stochastic rules. Initially, before individuals build up a travel history, each individual positioned at their “home airport” chooses a destination airport with probability proportional to the traffic flux [13], [19], 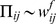. Since the flux matrix accounts for trips in which the individual remains under the same flight number, we allow for an agent choosing some other destination with a small probability, 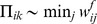.

The agent then establishes an itinerary, or space-time trajectory, to reach the destination. We make the ansatz that the route chosen minimizes a cost function, which generally increases with the cumulative time-in-transit and the monetary cost of the ticket. Given that the trip elapsed time correlates well with the number of connections and the physical travelled distance, and that ticket price decreases with route traffic, we use the following empirical cost function associated with origin 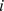 and destination 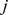:
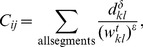
(1)where 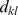 is the physical distance of the segment 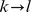 (accounting for the sphericity of the Earth), and the exponents 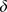 and 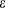 lie on the value ranges 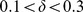 and 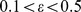. Which trip route is selected depends on the particular values of 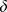 and 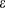. The ranges of values for these two parameters are chosen on the basis of producing itineraries that closely match those from real itinerary data [26]. To incorporate in our model the uniqueness of each passenger’s needs, we choose a unique combination of these two exponents for each individual. This reflects the current endemic heterogeneity in route selection from the wide range of connections, airline and price choices.

When an agent is off ground, we assume he moves between airports with a constant velocity of 650 km/h. When not flying, an agent can be at one of three distinct places: at their home node, at a connecting airport, or at a destination. The waiting times of an individual at each of these locations is clearly very different. We obtain waiting time distributions for connecting airports and final destinations from the individual mobility dataset [26], which indeed reflect a very different mean waiting time: in the order of a few hours at connecting airports, and a few days at destinations (Fig. 1*b*). Since the dataset lacks individual travel history, we cannot extract waiting times at the home airport, and we assume they are normally distributed [10], [17] with mean 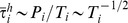 and standard deviation 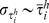, which recognizes that the average person in densely populated areas travels more often. This is based on the empirical relation between total traffic and population of an area [27]. For simplicity, we truncate the home waiting time distribution from below at 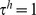 day.

An important aspect of our empirical model is the stochastic pattern of individual mobility that we implement. Initially, during a “training period” of 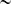
1 year, we let all agents choose destinations according to a traffic-weighted probability, as explained earlier (Fig. 1*c*). However, it is by now well established that individual mobility patterns are far from random [15] and that their statistics can be reproduced with two rules, exploration and preferential visit [16], which we introduce after the training period, once individuals have built some travel history (Fig. 1*d*). During exploration, an agent visits a new airport with probability 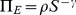, where 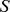 is the number of airports an agent has visited in the past. We use 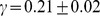 and 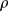 (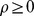) from a Gaussian distribution with mean 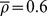 and standard deviation 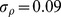, values that fit human mobility patterns from real mobile phone data [16]. In the absence of comprehensive data for individual long-range travel history, we make the assumption that the parameters used to reproduce local human mobility can be applied for long range travel. The new airport is chosen according to traffic from node 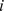. During preferential visit, the agent selects a previously-visited airport with complementary probability 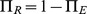. For an agent with home at airport 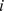, the probability 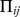 of visiting an airport 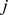 is proportional to the frequency 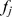 of previous visits to that location, 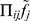. Because the travel history built by individuals is mediated by traffic, the mobility model with exploration and preferential visit honors the initial traffic flux matrix.

### Monte Carlo Simulations of Disease Spreading

For a single ‘mobility’ realization, we run our empirical model of human mobility through the air transportation network with 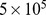 agents that are initially distributed in different “home” subpopulations. During an initial period of one year (training period), the agents are forced to choose destinations according to the traffic flux matrix. During this training period each individual develops a history of mobility patterns. Collectively, the mobility patterns honor the aggregate traffic structure from the dataset. During the second year, we incorporate the exploration and preferential-visit rules to assign destinations to individual agents. We use a time step of 0.5 hours, which we have confirmed is sufficient to resolve the temporal dynamics of the traffic-driven contagion process. For a given ‘mobility’ realization, we simulate the ‘reaction’ process as follows: we apply the SIR compartmental model at a randomly chosen time during the first half of the second year by infecting 10 individuals. In the study of late-time global attack, those 10 individuals are selected randomly across the entire network. For the study of early-time spreading, they are selected from the same subpopulation. For the Monte Carlo study, we average the results over 20 mobility and 200 reaction realizations.

### Reference Models

Our empirical model of human mobility through the air transportation network incorporates a number of dependencies that reflect the complex spatiotemporal structure of collective human dynamics. To understand which of these dependencies are essential, and which affect the modeling results to a lesser degree, we consider four different models of increasing complexity.

In *Model 1*, we consider the US air transportation network but retain only information about the topology of the network. We model mobility as a simplified diffusion process, in which all individuals perform a synchronous random walk, moving from one node to another, all at the same rate [10], [11]. We choose this rate to be the average rate at which individuals travel in our empirical model. Under these assumptions, all nodes with the same degree 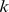 have the same behavior. We assign to each node a population corresponding to the stationary state, predicted by the mean-field theory [11]: for a node of degree 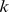, 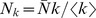, where 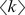 denotes the mean of the degree distribution 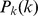, and 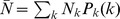 is the average nodal population.

In *Model 2*, we extend Model 1 by incorporating heterogeneity in the transition rates, as evidenced by the traffic data. To each node 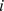 we assign a transition rate 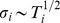, but individuals still select a destination randomly, with probability 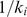.

In *Model 3*, we extend Model 2 by enforcing that destination selection by individuals is done according to traffic: the probability of an individual at node 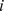 selecting destination 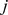 is proportional to 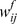.

In *Model 4*, we extend Model 3 by considering a simplified model of recurrent mobility patterns [17], [19]. Each individual is initially assigned to a “home” node. Individuals perform a random walk through the network of quenched transition rates and heterogeneous traffic, but return to their original subpopulation with a single recurrent rate 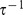 [17]. We select 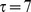 days, corresponding to the mean waiting time at destination airports obtained from actual data [26].

Several important differences exist between the reference models described above and our empirical model of human mobility. For instance, the reference models all discard geographic information. They also all assume that agent displacements are instantaneous and synchronous, taking place at discrete time integers (e.g. one day), and neglect the large heterogeneity in waiting times. We will see that resolving these spatio-temporal processes, while not critical for late-time measures of disease spreading, is essential in the early-time contagion dynamics.

### Global Attack

To study the dynamics of disease spreading through the air transportation network, we use the Susceptible–Infected–Recovered (SIR) contagion model. This model divides each subpopulation into a number of healthy (or susceptible, 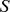), infected (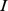) and recovered (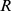) individuals, and it is characterized by a contagion reaction, 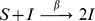, and a recovery reaction, 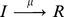, where 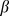 and 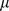 are the infection and recovery reaction rates, respectively, defined as the number of newly infected (resp. recovered) individuals per unit time for each initial infectious individual in a fully-susceptible subpopulation. Let 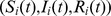 be the number of individuals in each class in node 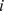 at time 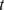, which satisfy 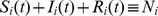 at all times. Under the assumption of homogeneous mixing within a city, the probabilities for a susceptible individual to become infected is 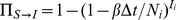, and for an infected individual to recover is 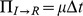, which reflect the dependence on the time step 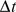. According to these rules, the expected increment in the infected and recovered populations at time 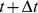 are 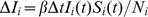 and 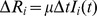, respectively, assuming that during the reaction step 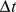 the subpopulation does not experience inflow or outflow of individuals. In our model, however, we track the state of each individual in the network. The reproductive number 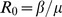 determines the ratio of newly infected to newly recovered individuals in a homogeneous, well-mixed and fully-susceptible population. From this observation follows the classic result on the epidemic threshold in a single population, 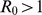. Much work has been devoted to the study of epidemic thresholds in metapopulation networks [10], [11], [17], which generally shows that the reproductive number must be greater than 1 for global spreading of an outbreak.

We apply the SIR contagion model to the four reference models described above and to our empirical mobility model. We employ the *global attack*, defined as the asymptotic (late-time) fraction of the population affected by the outbreak, as our measure of the incidence of the epidemic. We initialize the disease with a small number of infected individuals randomly chosen from the whole population. We obtain representative statistics by performing a Monte Carlo study and averaging over many realizations.

**Figure 2:**
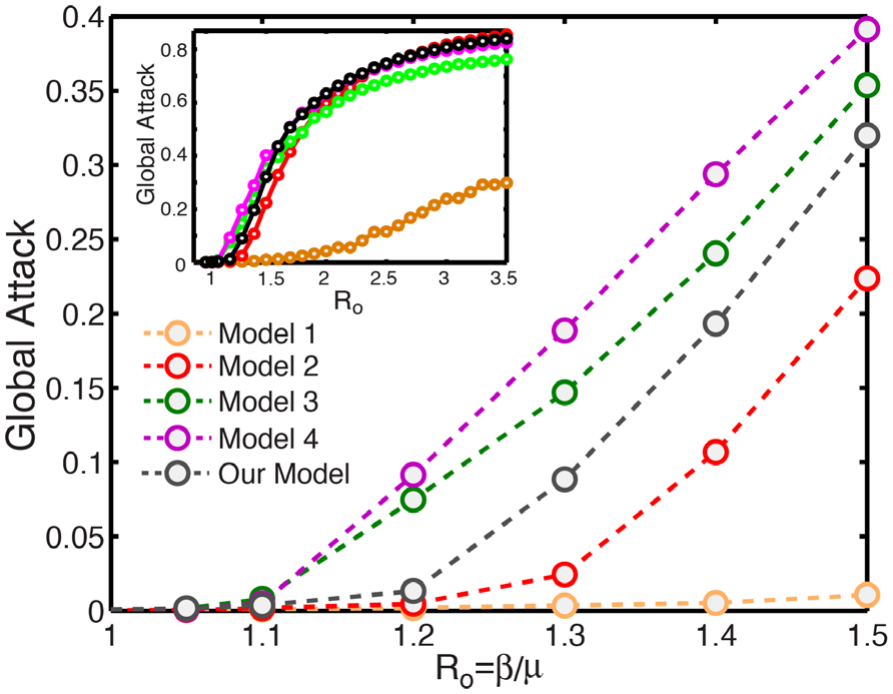
Monte Carlo study of the global attack of an epidemic as a function of the reproductive number 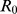, for the different models explained in the text. We used a value of the recovery rate 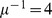 days. We initialized the epidemic with 10 infected individuals chosen randomly across the network. We used a population of 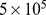 individuals, and average our results over 200 realizations. (Inset) The global attack for larger values of 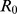 exhibits smaller differences among models, except for those between annealed and quenched transition rates at the nodes, as evidenced by the simulation results of Model 1 vs. the other models.

We find that the global attack is quite sensitive to the degree of fidelity of the metapopulation mobility model, especially in the range of low reproductive numbers (Fig. 2). Naturally, the global attack increases with 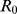 for all models. There is a dramatic difference in the global attack between Models 1 and 2, highlighting the critical influence of quenched disorder in the transition rates 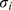 out of individual subpopulations. The global attack increases also from Model 2 to Model 3, reflecting the super-diffusive anomalous nature of spreading when agent displacements are driven by traffic, as opposed to a diffusive random walk [12], [13]. In comparison with these two effects––quenched disorder in transition rates and traffic-driven spreading––recurrent individual mobility patterns [17], [19] have a relatively mild influence on the global attack, as evidenced by the differences between Models 3 and 4. We observe that the additional complexity included in our empirical model––geographic information, high-fidelity individual mobility, and time-resolved agent displacements––induces a slight *delay* in the epidemic threshold with respect to Models 3 and 4, indicating the nontrivial dependence of contagion dynamics on human mobility.

### Influential Spreaders

Finding measures of power and centrality of individuals has been a primary interest of network science [28], [29]. The very mechanism of preferential attachment shapes the growth and topology of real-world networks [1], indicating that the degree of a node is a natural measure of its influence on the network dynamics. Another traditional measure of a node’s influence is the betweenness centrality, defined as the number of shortest paths that cross through this node [28]. Betweenness centrality does not always correlate strongly with the degree, the air transportation network being precisely an example of poor correlation between the two [23]. It has been shown, however, that certain dynamic processes such as SIS or SIR epidemic spreading in complex networks appear to be controlled by a subset of nodes that do not necessarily have the highest degree or the largest betweenness [24].

Here we revisit what is meant by spreading, and make a crucial distinction between the asymptotic *late-time* behavior––which has been studied more extensively––and the *early-time* dynamics, for which much less is known. We show that the two behaviors are controlled by different mechanisms and, as a result, require different measures of spreading.

#### Influential spreaders at late times

**Figure 3:**
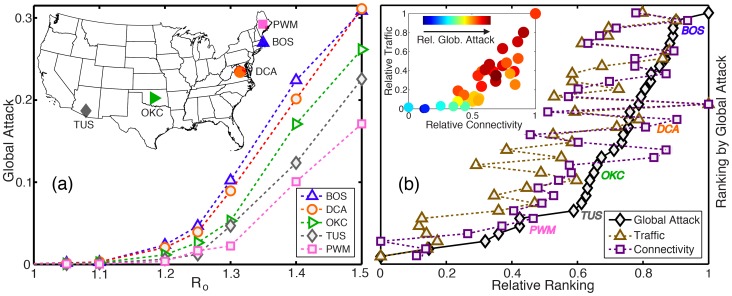
Late-time spreading ability of different airports, measured by the global attack of an SIR epidemic that originates at each airport. (*a*) Global attack as a function of reproductive number, for five different airports (see inset). We initialize the disease by infecting 10 randomly chosen individuals inside the subpopulation of consideration. We use 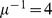 days. Each point is the result of a Monte Carlo study averaging over 200 reaction and 20 mobility realizations and using 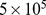 individuals. (*b*) Ranking of the 40 major airports in US in terms of their spreading ability measured by the normalized global attack. We compare the normalized global-attack ranking curve (black diamonds) to the ones that result from considering the airport’s normalized degree (magenta squares) and the airport’s normalized traffic (brown triangles). Also shown is the ranking of the airports shown in (*a*). Both degree and traffic provide effective rankings of influential late-time spreaders, which in this case can be understood from the good cross-correlation between the two (inset).

We perform numerical simulations of epidemic spreading in our model by initializing the SIR compartmental model with infectious individuals at one single subpopulation. We compare the asymptotic, late-time spreading ability of different subpopulations by means of the global attack of the SIR epidemic (Fig. 3*a*). We study low values of the reproductive number 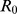, between 1 and 1.5, because the relative differences among different sources of infection are largest in this limit. Recent outbreaks of influenza A are estimated to lie within this range [30]. We rank the 40 major airports in the United States in terms of their asymptotic global attack, after aggregating the ranking over the range of reproductive numbers studied (Fig. 3*b*). The ability of a node to spread an epidemic depends on fast dispersal of agents to many other nodes, thereby increasing the probability of infectious individuals contacting a large population before they recover. Thus, intuitively, the asymptotic spreading ability of a node increases with its traffic and connectivity. In fact, we find that both degree and traffic provide fair rankings of influential late-time spreaders because in the air transportation network both quantities are strongly correlated (Fig. 3*b*, inset).

#### Influential spreaders at early times

Late-time measures of spreading, such as the asymptotic global attack, cannot capture the details of early-time contagion dynamics. The vigor of initial spreading, however, is likely the crucial aspect in the assessment and implementation of remedial action for highly contagious diseases [7], when the reaction and transport timescales are comparable.

The natural measure of physical spreading is the *total square displacement* (TSD) of the infected agents,
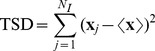
(2)where 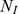 is the total number of infected individuals at time 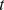, 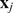 is the position of the infected individual 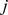, and 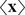 denotes the position of the center of mass of infected individuals. The TSD increases with time as the infected agents, initially all in the same node, spread through the air transportation network by traffic and contact individuals at the connecting and destination nodes.

We compare the TSD for 40 major airports in the US, 10 days after the infection starts at each of those airports, and a reproductive number 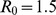. The random walk described by the infected agents is asynchronous (heterogeneous travel times and waiting times), traffic-driven (quenched disorder in the network fluxes), non-Markovian (recurrent individual mobility patterns) and non-conservative (appearance and disappearance of infected agents due to infection and recovery). This complexity requires that the transport and contagion processes be time-resolved, an essential feature of our model.

**Figure 4:**
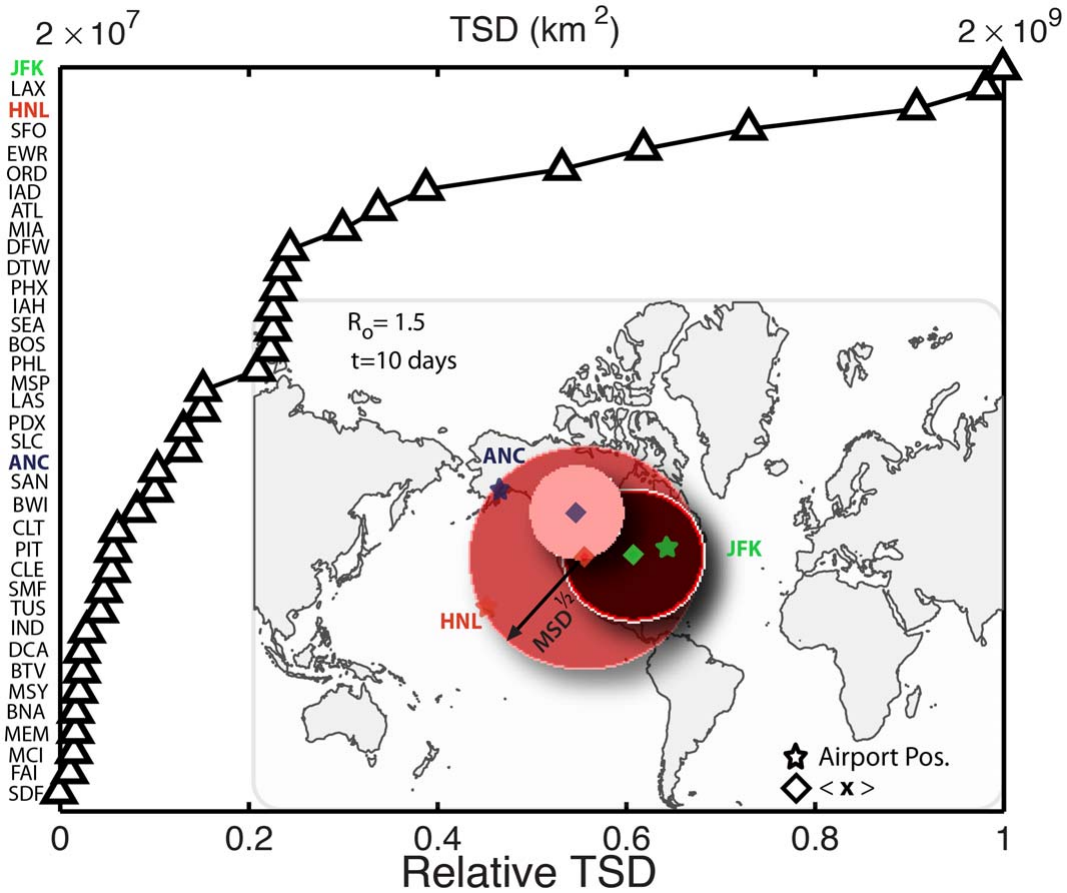
Ranking of influential spreaders by the normalized early-time mean square displacement of infectious individuals. We initialize the disease by infecting 10 individuals from each specific airport (see inset), and use 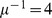 days. Each point is the result of a Monte Carlo study averaging over 100 reaction and 20 mobility realizations and using 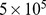 individuals. (Inset) Graphical representation of the mean position of infected individuals, 10 days after the outbreak from three different locations. The circle radius denotes the geographic extension of the infectious cloud (as measured by the square root of the Mean Square Displacement [12] of infected individuals) while their color represents the number of infected at the same time (dark colors denote large number of infected).

We rank all 40 airports according to their TSD at early times. The curve of ordinal ranking vs. normalized TSD is markedly concave, indicating that only a handful of airports are very good spreaders (Fig. 4). The list of early-time super-spreaders is led by J. F. Kennedy (JFK), Los Angeles International (LAX), Honolulu (HNL), San Francisco (SFO), Newark Liberty (EWR), Chicago O’Hare (ORD) and Washington Dulles (IAD).

**Figure 5:**
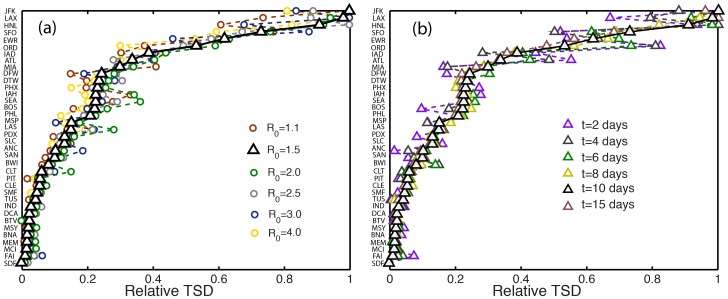
Ranking of influential spreaders by the normalized early-time Total Square Displacement. (*a*) for different reproductive numbers, 10 days after the disease is initiated. (*b*) at different times after the initiation of the disease. We use 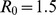 and 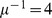 days. Each point in the above plots is the result of a Monte Carlo study averaging over 100 reaction and 20 mobility realizations and using 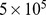 individuals.

We perform a sensitivity analysis with respect to the reproductive number, 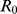, and the number of days after which the TSD is measured (Fig. 5). Clearly, a higher reproductive number leads to a more aggressive spread of the disease, and therefore larger values of the total square displacement at the same time. From its definition, it is also clear that the TSD increases with time, at least until saturation. Importantly, while the absolute value of TSD depends strongly on the 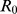 and the time of calculation, the ranking of influential spreaders according to TSD appears to be rather insensitive to these parameters, at least for times in the order 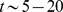 days (Fig. 5*b*).

**Figure 6:**
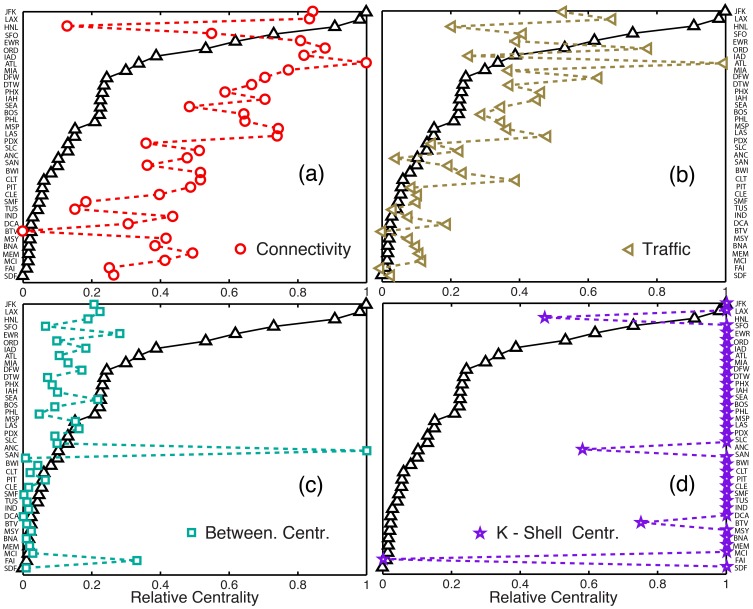
Ranking of influential early-time spreaders by existing metrics. Shown are the results from the model simulations (black triangles), and comparison with the ranking provided by existing metrics of centrality and late-time influential spreading. (*a*) Normalized degree. (*b*) Normalized traffic. (*c*) Normalized betweenness centrality. (*d*) Normalized 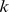-shell centrality.

It is instructive to compare the TSD-ranking curve with the rankings provided by existing metrics of centrality and influential spreading, including the normalized degree [1] (Fig. 6*a*), traffic (Fig. 6*b*), betweenness centrality [23] (Fig. 6*c*) and 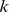-shell centrality [24] (Fig. 6*d*). Similar results to those from total traffic are obtained with the eigenvector centrality of the weighted mobility matrix (not shown). All of these metrics deviate significantly from the empirical simulations. For instance, HNL causes large physical spreading, even though it is the airport with the second lowest number of connections, and its traffic is only 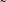
20% of that of Atlanta International (ATL). Equally surprising is that ATL has *both* the largest degree and the largest traffic, yet it comes in 8th place, with an early-time spreading power as low as 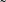
30% that of the best spreader (Fig. 6*a,b*). Betweenness centrality is able to identify the poor spreaders, but does not provide accurate ranking or spreading power among the good ones (Fig. 6*c*). For example, Anchorage International (ANC) has the largest betweenness centrality, yet it ranks low as an early-time spreader. The 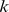-shell centrality, which has recently been proposed as an effective metric for identifying influential spreaders at late-time [24], gives no information about early-time spreading (Fig. 6*d*).

#### Geographic spreading centrality

**Figure 7:**
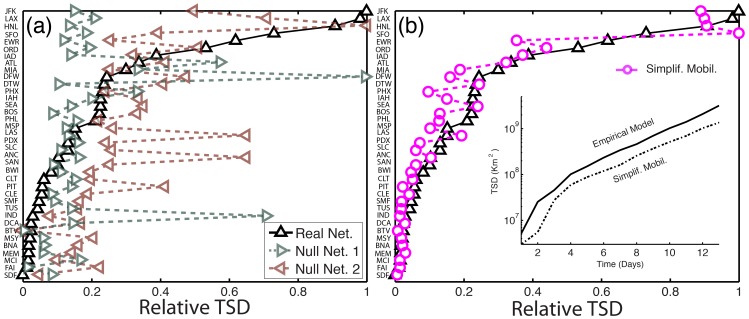
Role of spatial organization, traffic quenched disorder, and mobility patterns, on early-time spreading. (*a*) Shown is the TSD-ranking for individual realizations of two null networks testing the influence of (1) geographic locations of the nodes, and (2) heterogeneity in the traffic of the links. The dissimilarity between those rankings and that from the original network model strongly suggests that any effective measure of influential early-time spreaders must incorporate geography and traffic quenched disorder. (*b*) TSD-ranking for a simplified model of human mobility. Removing the detailed patterns of mobility affects the evolution of the predicted TSD (see inset for HNL airport) but does not affect the early-time spreading ranking significantly.

It is clear that existing metrics of influential spreading do not properly capture the early-time spreading behavior. We hypothesize that the main reason for this disparity is that they do not account for geographic information and the network’s traffic spatial organization. To test this hypothesis we develop two null networks. As opposed to the reference models presented earlier, which were introduced to incorporate an increasing degree of realism and identify key factors affecting the late-time global attack, the null *networks* employ the *same* empirical model, but modify specific aspects of the network to test whether they have an important bearing on early-time spreading. Null network 1 has the same degree and traffic distributions as the original air transportation network, but changes the geographical information by randomizing the identity of the nodes. In null network 2, we eliminate the traffic quenched disorder by homogenizing outgoing probabilities across the nodes’ links, but preserving the position of the nodes. We apply the same mobility and epidemic models and we rank the same airports according to TSD. We find that these rankings are always, for each realization of the null networks, profoundly dissimilar to that of the original network (Fig. 7*a*). This confirms the importance of the geographic location of airports, which affects spreading directionality, and the importance of traffic heterogeneity, which affects the routing dynamics, suggesting that both spatial relations and traffic structure are critical elements in early-time spreading.

We also performed a comparison between the detailed empirical model and a model that is identical in all aspects except in that it employs a simpler mobility model. In the simplified model, all agents behave statistically in the same way, with no travel history and with a single return rate (equal to the inverse of the mean waiting time at destinations). The choice of destination from a given origin is random, weighted by traffic from the origin-destination database. A constant time step 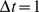 day is used, therefore removing the detailed mobility dynamics. We find that, while the evolution of the TSD does depend on the details of the mobility model, the ranking of spreading power exhibits little dependence (Fig. 7*b*), suggesting that individual mobility patterns can be neglected in the construction of a simple metric of influential spreading.

In the light of these observations, we propose a new metric to characterize the ability of an airport to spread an infection spatially at early times, the *geographic spreading centrality* (GSC). We express the vector of airport spreading centralities 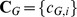 as.
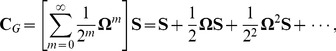
(3)where 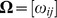 is the normalized traffic flux matrix, with 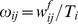, and where 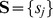 is the vector of airport *spreading strengths* [31], defined as



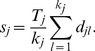
(4)The spreading strength is a *local* measure that accounts for the node’s traffic, degree, and spatial scale of influence. The overall spreading ability of a node, however, must reflect the spreading strength of its neighbors, its neighbors’ neighbors, and so on. This notion has led to the classical understanding of the centrality of a node as a generalized eigenvalue problem [29], from which our definition of GSC in Eq. 3 follows naturally.

**Figure 8:**
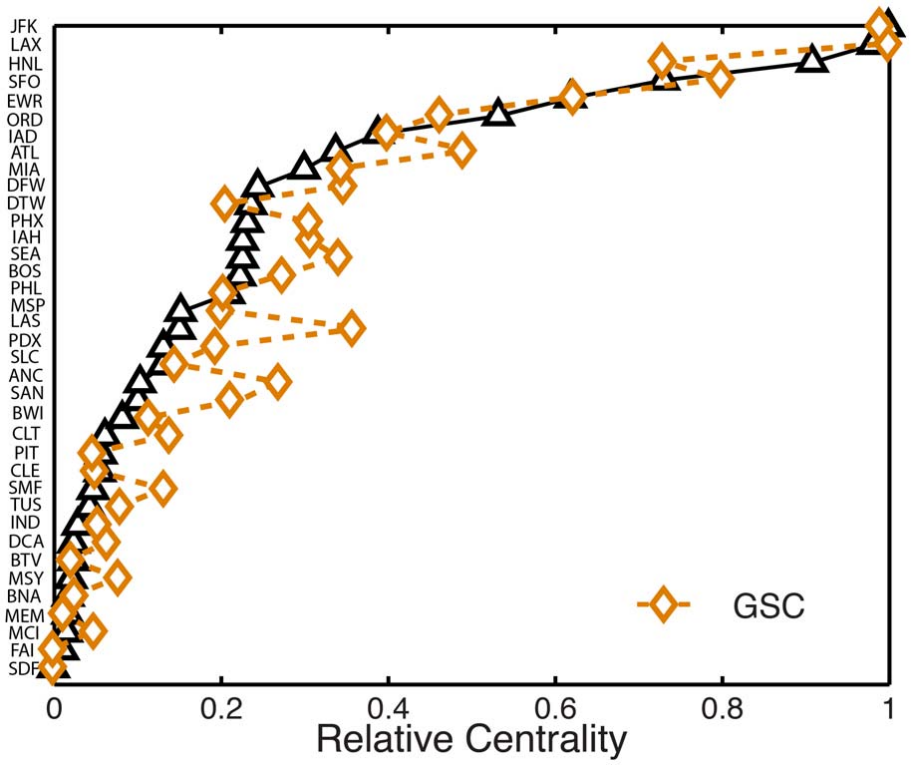
Ranking of influential spreaders at early times from the geographic spreading centrality (GSC). The GSC metric predictions are in quantitative agreement with the results from the Monte Carlo study on the empirical model.

We compare the airport rankings predicted by GSC with those obtained from the model simulations, and find excellent quantitative agreement (Fig. 8), suggesting that GSC is a reliable *a priori* metric of influential early-time spreaders.

To quantify the correlation between the ranking provided by the TSD and the centrality measures, we use the Kendall tau (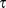) rank coefficient [32]. This correlation coefficient indicates how rankings from two quantities are qualitatively correlated and takes a value of 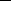
1 if the two rankings are negatively correlated, 0 if the two rankings are independent, and 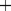
1 if they are positively correlated. The correlation coefficient of the rankings by TSD and connectivity (Fig. 6a) is equal to 0.53, by TSD and Traffic (Fig. 6b) is equal to 0.57, by TSD and betweenness centrality (Fig. 6c) is 0.48 and by TSD and k-shell centrality (Fig. 6d) is 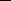
0.02. The ranking by the proposed centrality (GSC) and by TSD (Fig. 8) are correlated with a Kendall tau of 0.87.

It is worth discussing the spreading power of specific airports in the light of the GSC ranking. Classical measures of centrality, such as total traffic or connectivity, would suggest that Atlanta International airport (ATL) would have the largest spreading ability. This is clearly not the case, as it ranks 8th in terms of spreading power. The reason is that much of that traffic is of regional nature, within North America, and that many of the connected airports are not, themselves, strong spreaders. The GSC metric allows for a rationalization of the surprising fact that an airport like Honolulu (HNL) ranks third in early-time spreading, very close to JFK and LAX. Despite having a relatively low connectivity (Fig. 6*a*) and total traffic (Fig. 6*b*), HNL combines three important features that catalyze contagion spreading: (1) it is dominated by long-range travel; (2) it is well connected to other massive hubs, which are themselves powerful spreaders; and (3) it is geographically located such that East-West travel is balanced, thereby maximizing TSD growth. Importantly, these aspects are all captured in the definition of the geographic spreading centrality (Fig. 8).

## Discussion

Characterizing the early-time behavior of epidemic spreading is critical to inform decisions during public-health emergencies, and to design regulations aimed at mitigating global pandemics. Here, we show that subpopulations that act as powerful spreaders of infectious diseases at early times––identified by the TSD during the first 10 days of the contagion–– differ significantly from the central spreaders in terms of the late-time global attack.

Simulating the infectious dynamics during the initial stages of spreading requires a modeling framework in which transport and contagion processes are time-resolved. We develop a stochastic-agent mobility model through the air transportation network that relies on 3 years of US-centric air travel data and four months of individual travel itineraries. We use this database to build empirical distributions of waiting times at connecting airports and final destinations, and train the model to reproduce the recurrent mobility patterns of individuals. Our analysis demonstrates that the detailed spatiotemporal signatures of individual mobility patterns collectively impact epidemic spreading, especially in the range of low reproductive numbers.

Existing metrics of influential spreaders in networks were not designed to characterize the early-time spreading behavior. Here we propose a new metric, the geographic spreading centrality, which accounts for the local strength in terms of the node’s traffic, degree and spatial scale of influence, as well as its global role within the network by incorporating the strength of its neighbors. This metric is able to successfully rank influential spreaders at early times, as evidenced by the agreement between the metric’s prediction and detailed Monte Carlo simulations. The geographic spreading centrality opens the door to the quantitative understanding of spreading dynamics on other networks embedded in space, in which topology alone is insufficient to fully characterize the system [33].
